# A Mozart is not a Pavarotti: singers outperform instrumentalists on foreign accent imitation

**DOI:** 10.3389/fnhum.2015.00482

**Published:** 2015-08-28

**Authors:** Markus Christiner, Susanne Maria Reiterer

**Affiliations:** ^1^Department of Linguistics, Unit for Language Learning and Teaching Research (SLLF), University of ViennaVienna, Austria; ^2^Centre for Teacher Education, University of ViennaVienna, Austria

**Keywords:** vocal motor system, memory, speech imitation, language acquisition device, singing ability, instrumentalists versus vocalists, vocal flexibility

## Abstract

Recent findings have shown that people with higher musical aptitude were also better in oral language imitation tasks. However, whether singing capacity and instrument playing contribute differently to the imitation of speech has been ignored so far. Research has just recently started to understand that instrumentalists develop quite distinct skills when compared to vocalists. In the same vein the role of the vocal motor system in language acquisition processes has poorly been investigated as most investigations (neurobiological and behavioral) favor to examine speech perception. We set out to test whether the vocal motor system can influence an ability to learn, produce and perceive new languages by contrasting instrumentalists and vocalists. Therefore, we investigated 96 participants, 27 instrumentalists, 33 vocalists and 36 non-musicians/non-singers. They were tested for their abilities to imitate foreign speech: unknown language (Hindi), second language (English) and their musical aptitude. Results revealed that both instrumentalists and vocalists have a higher ability to imitate unintelligible speech and foreign accents than non-musicians/non-singers. Within the musician group, vocalists outperformed instrumentalists significantly. Conclusion: First, adaptive plasticity for speech imitation is not reliant on audition alone but also on vocal-motor induced processes. Second, vocal flexibility of singers goes together with higher speech imitation aptitude. Third, vocal motor training, as of singers, may speed up foreign language acquisition processes.

## Introduction

Recent research has shown that musical expertise heightens the potential to memorize and reproduce foreign languages orally (Nardo and Reiterer, 2009; Reiterer et al., 2011; Hu et al., 2012; Christiner and Reiterer, 2013). This relatively newly established and steadily growing scientific field, however, has hardly ever differentiated between two, for the faculty of language learning, relevant aspects: (1) different kinds of musical aptitudes such as instrument playing vs. singing; and (2) language testing of intelligible and unintelligible utterances.

While for measuring musical expertise already approved tests are available (e.g., Advanced Measures of Music Audiation, AMMA; Gordon, 1989), measuring speech imitation talent/aptitude remains a complex endeavour as individual differences in language production and perception can even be noted in native speakers (Pakulak and Neville, 2010; Andringa, 2014) and giftedness, as raw material, is considered to be a natural and inherent ability, free of educational influence (Gagne, 2005). This predetermines speech imitation test design and makes unintelligible and geographically and linguistically distant languages ideal test stimuli for defining an individual’s speech imitation aptitude.

In language acquisition, accent imitation is sometimes considered one of the most crucial aspects of L2 learning. For example, already Seliger (1978) proposed that there are many critical periods for different aspects of language, with the ability to master a native accent in a foreign language being the first to be lost, around the onset of puberty. Hence, the aspect of “phonetic ability” has often been considered the first or only sub-ability in language learning which is ultimately subjected to a critical period (Moyer, 2014). Furthermore, pronunciation/accent ensures adequate communication and illustrates the speaker’s proficiency (Dalton-Puffer et al., 1997). Sounding like a native speaker is a high aim a second language speaker wants to achieve. For the language learner, foreign accent imitation can be a challenging task, as languages differ on multiple aspects and even typologically close languages such as English and German, have many language specific consonants, vowels or diphthongs, causing non-native speakers difficulties with pronunciation. Typologically more distant languages such as South Asian languages (e.g., Hindi) convey phonetic contrasts such as retroflex vs. dental stop distinctions which are scarcely generated within European languages (Werker and Tees, 2005). Along with this, languages vary on their syllabic rhythm patterns dividing languages into stress-timed, syllable-timed and mora-timed languages. Hindi, for instance, has been classified as syllable-timed language, while German has been termed stress-timed. Characteristically, non-natives face difficulties to discriminate and generate unusual non-native contrasts (Werker et al., 1981; Tees and Werker, 1984; Polka, 1991; Polka et al., 2001; Werker and Tees, 2005) and most often fail to understand where a word begins or ends in a speech stream (Patel, 2008). This may go some way with ageing as language specification of the mother tongue goes at the expense of plasticity towards the acquisition of new languages in most adults.

This is not fully true for musicians who are equipped with special skills and imitation abilities. Many recent studies reported a significant relation between speech imitation and musical expertise. Musicians always outperformed non-musicians in language imitation tasks (Schön et al., 2004; Thompson et al., 2004; Wong and Perrachione, 2007; Pastuszek-Lipinska, 2008; Milovanov, 2009; Nardo and Reiterer, 2009; Reiterer et al., 2011; Hu et al., 2012). As far as known, studies hardly ever differentiated between musical “sub-abilities” and investigated whether the imitation skills of instrumentalists and vocalists contribute differently or not.

In previous investigations on language aptitude, singing capacity and musical instrument playing yielded strong correlations to the ability to imitate speech (Nardo and Reiterer, 2009; Reiterer et al., 2011). In a follow up behavioral experiment on vocalists earlier results were replicated and singing capacity contributed significantly to the imitation of familiar and unfamiliar utterances, but surprisingly perceptual musicality measurements correlated significantly lower with speech imitation skills and were irrelevant for explaining participants’ imitation abilities in regression models (Christiner and Reiterer, 2013). Singers’ musical abilities may be based on different skills than those developed by or pre-existent in instrumentalists. Most investigations focus on a purely perceptual advantage of musicians’ language capacity and ignore the role of production. In the present study we integrated both.

Pre-speech studies have shown that the melodic elements of infants’ cry might form the basis for both music and speech (Wermke and Mende, 2006, 2009) and may derive from one source which develops into separately, but closely related faculties later. On the perceptual and structural level language like music consists of rhythmic cues, language prosody. Music’s metrical organization resembles that of language as beat, notes and “patterns of tense and relaxation” form higher units (Jackendoff and Lerdahl, 2006). Language prosody, for example, organises speech and consists of various elements such as “tonal, temporal and dynamic features” (Trofimovich and Baker, 2006). Prosodic variations of oral language also “share many acoustic features with tone transitions in musical melodies” (Oechslin et al., 2010). The ability to discriminate temporal or segmental information requires similar processes for perceiving both, speech and music. Brain imaging studies found that prosodic information has more strongly activated musical associated areas on the right hemisphere (Meyer et al., 2002), especially when linguistic information is reduced (Perkins et al., 1996). Evidently, music and language perception is not an either/or choice but highly interconnected and may be one of the underlying mechanisms why musicians are advantaged in the oral acquisition of foreign languages: musical expertise leads to an improvement of both, music and speech perception (Oechslin et al., 2010) but also to enhanced literacy and attentional skills (Seither-Preisler et al., 2014).

### Neuronal Underpinnings of Vocalisations

What has widely been ignored so far is that vocalists train their vocal apparatus more precisely than instrumentalists do. Speech production is closer to the nature of singing than the nature of instrument playing. Hence, a singer’s instrument is integrated into the body and already used for all forms of vocalization. Considering this, singing and instrument playing could be understood as separate musical abilities, especially when comparing musical expertise to language learning tasks.

Generally speaking, all forms of vocalization are based on the same principles and rely on the integration of multiple networks. While speech perception has been considered to be more left hemisphere dominated (Liégeois-Chauvel et al., 1999), vocal sound perception seems to be largely bihemispherically organized “along the upper bank of the superior temporal sulcus” regardless of whether participants were exposed to speech or non-speech sounds (Belin et al., 2000). All forms of vocalization such as song and speech require the control of the laryngeal system and the articulatory apparatus such as tongue, jaw and orofacial muscles (e.g., Zarate, 2013). Most of the former mentioned systems are largely bihemispherically organized. Orofacial and supralaryngeal movements show a largely bihemispherically organized specialization (Özdemir et al., 2006; Grabski et al., 2012a,b). This also applies to laryngeal processes. The neural correlates of the supralaryngeal movements include the “sensorimotor cortex […], the supplementary motor area and the superior celebellar hemispheres” (Ackermann, 2008; Grabski et al., 2012a) on both hemispheres as well as orofacial motor control in the central sulcus, rostral region of the precentral gyrus and the caudal areas of the postcentral gyrus bilaterally (Grabski et al., 2012b). Zarate (2013) proposes that, regardless of whether utterances are sung or spoken, vocalization relies on “M1, ACC, basal ganglia, thalamus, and the cerebellum”. In addition, vocalization requires the integration of auditory cortex, insula, parietal and premotor regions and the integration of somatosensory feedback of the primary and secondary somatosensory cortex (Ackermann, 2008; Ackermann and Riecker, 2010; Zarate, 2013; Ziegler and Ackermann, 2013; Ackermann et al., 2014). A comparative study on singing vs. speech found bilateral activation in the inferior pre- and postcentral gyrus, the superior temporal gyrus (STG) and the superior temporal sulcus during singing and speech production (Özdemir et al., 2006). However, singing, in marked contrast to speech production, shows further activation in the primary sensorimotor cortex and in the mid-portions of the STG (Özdemir et al., 2006). Speaking and singing share large parts of neural correlates suggesting that singing training, more than instrument playing, leads to higher language imitation abilities. This has been corroborated more recently in discrimination training and testing, suggesting that auditory training alone does not improve vocal accuracy in non-musicians (Zarate et al., 2010). Behavioral investigations, on the other hand, came to a similar conclusion demonstrating that changing vocal commands of specific utterances leads to perceptual shifts (Nasir and Ostry, 2009). In the light of the present findings it could be assumed that vocalists develop different networks when compared to non-musicians and instrumentalists.

### Neural Correlates of Musicians vs. Singers

Neuroscientific evidence has shown that musicians show alterations of their brain structure when compared to non-musicians (e.g., Schneider et al., 2002, 2005; Gaser and Schlaug, 2003; Seither-Preisler et al., 2014). The alterations of musicians’ brain structure have been explained to take place during specific conditions achieved through musical training. The OPERA hypotheses, proposed by Patel (2011), claims that five conditions are essential to impose music induced neural plasticity. The model suggests that: (a) overlaps of speech and music processing; (b) more precisely processing of music in general; (c) positive emotions of music; (d) musical activities which are repetitive in their nature; and (e) focused attention taken together should lead to benefits in speech processing. Vocalists, on the other hand, have to train one more condition which can be differentiated from the more general musical training, their vocal motor apparatus.

Studies focusing on the difference between instrumentalists and vocalists are scarcely conducted as it is a challenging task to find participants. An interesting study conducted by Schneider et al. (2005) focused on contrasting musical abilities. They found individual differences in pitch perception even within vocalists where sopranos showed higher fundamental pitch discrimination ability in marked contrast to altos. They concluded that the size of the neural Hesch’s gyrus depends on the musical ability. Musicians, that is to say instrumentalists and vocalists, show anatomical alterations of the gray matter (Schneider et al., 2002), the Heschl’s gyrus (Schneider et al., 2002, 2005; Gaser and Schlaug, 2003; Seither-Preisler et al., 2014), and the arcuate fasciculus (Halwani et al., 2011). More recently, Halwani et al. (2011) compared musicians to non-musicians, and vocalists. Results revealed that vocalists show additional structural adaptations in the arcuate fasciculus which have not yet been found in pure instrumentalists (Halwani et al., 2011). This suggests that vocal-motor training induces a change in the volume and complexity of white matter tracts (Halwani et al., 2011). These adaptations seem to improve the interplay between the auditory feedback system and the kinesthetic system (Kleber et al., 2010). Trained singers can rely more on somatosensory feedback compared to non-singers and instrumentalists. At the other extreme, our closest relatives, monkeys, lack a complex connectivity between the auditory system and the oromotor system (Rilling et al., 2012; López-Barroso et al., 2013) which might be one explanation why monkeys are unable to store rapidly occurring acoustic signals, although they have a high proficiency in mastering tactile and visual recognition (Schulze et al., 2012). Schulze et al. suggest that “… in audition alone, monkeys seem unable to store stimulus representations” concluding that the oromotor system assists memorization of speech sounds in humans. If the oromotor system is involved in memorization of speech signals, we hypothesize that vocalists with their refined vocal motor ability will outperform instrumentalists when imitating new foreign speech material. This would be additional evidence that: (A) human’s vocal motor system is involved in laying down memory of utterances; and (B) show that vocal motor training should become an integral part of language learning settings (Fonseca-Mora et al., 2011).

## Materials and Methods

### Speech Imitation: Hindi Stimuli

For testing the speech imitation skills of the participants we used Hindi stimuli which have already been tested in previous investigations (Nardo and Reiterer, 2009; Reiterer et al., 2011; Hu et al., 2012; Christiner and Reiterer, 2013). The participants had to repeat four simple sentences (statements) of equal syllable length (11 syllables) which consisted of either five or six words. The Hindi sentence material contained difficult retroflex consonants within 11 syllables: retroflex *n* and retroflex *r*. Retroflex consonants are both difficult to perceive and to produce for German native speakers. The correct articulation requires the tongue curled back against the palate. Additionally, Hindi is syllable-timed and rhythmically differently organised compared to the participants’ mother tongue and aims at measuring the potential of a person’s speech imitation aptitude by excluding all educational influence/benefits.

The recording was performed in a soundproof room where the participants were introduced to their tasks. For the familiarisation task the participants were listening to Hindi stimuli three times via loudspeakers (*ADAM A7*) and were asked to repeat the sentences after the third time without recording. After the familiarisation task, the participants used studio headphones (*BEYERDYNAMIC DT-770 PRO*) and repeated the Hindi stimuli in the best accent they could manage, while being recorded. The recordings were rated by seven naïve Hindi native speakers who evaluated the overall performance of “sounding like a native Indian” on an intuitive rating scale from 0 (min) to 10 (max) with no particular reference to individual phonetic characteristics. To ensure that raters understood their task correctly, we instructed them to think of characteristics such as word stress, the rhythm of the language, intelligibility and pronunciation.

### Musicality Test: AMMA

The *AMMA* test measures the participants’ perceptual musical abilities. The test consists of 30 musical statements where the participants have to discriminate tonal and rhythmical changes of two musical statements or indicate that they are the same (Gordon, 1989). The AMMA test can be targeted to high school students and university music and non-music majors. For this task the participants performed the familiarization task which consists of three conditions. Either the paired musical statements were the same, included a tonal or a rhythmic change. After familiarization, the participants used headphones (*BEYERDYNAMIC DT-770 PRO*) and were asked to perform the task within a single sitting and decide whether the paired musical statements were the same, included a tonal or a rhythmical change.

### Participants

A sample of 96 participants was selected and comprised 33 vocalists, 27 instrumentalists and 36 non-singers/non-musicians (67 female and 29 male participants) of a large age range (20–59). None of the participants reported any hearing problems or other impairments and gave informed consent. The participants were selected according to their musical abilities and their language knowledge. Choosing Hindi for testing German native speakers was based on the fact that German native speakers are largely unfamiliar with this particular language. The participants were asked to inform whether they speak Hindi or not. Furthermore, people who were exposed to this language were excluded from the research. This should ensure that participants have had no experience in Hindi before. The participants were all monolingual German native speakers. All participants reported to speak English. 59.4% of the participants additionally spoke French, followed by 32.3% who spoke Spanish and 22.9% who mastered Italian. 25.0% of them spoke other languages. Further criteria were that vocalists did not play instruments professionally and defined themselves as singers. Additionally, the vocalists received formal vocal education for at least 2 years and were rated and defined as advanced singers by professional singing educators. The instrumentalists chosen played one or two instruments actively at an excellent level and had no history of vocal instruction or semi-professional singing activities (hobby singing). The control group had no experience in singing apart from traditional singing activities such as happy birthday, and only little experience in instrument playing at a very basic level and they defined themselves as non-musicians. The strict participants’ selection aimed at dissociating instrumentalists from vocalists and non-musicians/non-singers from musicians in general.

## Results

### Data Analysis

For analyzing the results, we ran one-way ANOVAs. The first one-way ANOVA was performed to find out whether the speech imitation ability in Hindi of the instrumentalists, vocalists and the non-musicians shows differences. The second one-way ANOVA was performed to analyze whether the musical ability to discriminate rhythmical and tonal differences shows significant differences between the instrumentalists, musicians and non-musicians. Age, gender and the number of languages spoken had no effect on the musicality test or speech imitation tasks.

### Results Speech Imitation ANOVA

The mean of Hindi imitation for vocalists was 4.35, *SD* = 1.15, for instrumentalists 2.28, *SD* = 1.31, and for non-musicians/non-singers 1.56, *SD* = 1.20.

**Figure 1:**
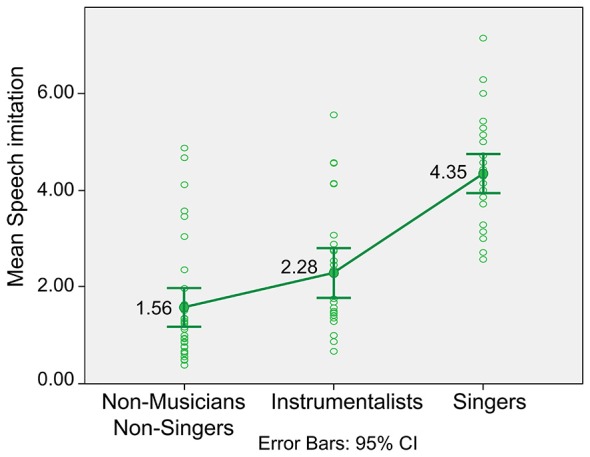
**ANOVA Hindi speech imitation.** This figure shows the differences in the performance of Hindi of non-musicians/non-singers, instrumentalists and vocalists (singers). Instrumentalists were significantly better than non-musicians/non-singers but singers were significantly better compared to both, non-musicians/non-singers and instrumentalists.

1. Musical expertise shows a significant influence on Hindi imitation proficiency, *F*_(2,93)_ = 47.98, *p* < 0.01, *ω* = 0.70.
2. Planned contrasts revealed that vocalists are significantly better speech imitators compared to instrumentalists *t*_(93)_ = 6.58, *p* < 0.01 (one-tailed), and to non-musicians/non-singers *t*_(93)_ = 9.54, *p* < 0.01 (one-tailed). Further contrasts indicated that instrumentalists are also significantly better speech imitators compared to non-musicians/non-singers *t*_(93)_ = 2.32, *p* < 0.05 (one-tailed; see Figure 1).

### Results Musicality Test ANOVA

The mean of the musicality test for vocalists was 60.27, *SD* = 8.21, for instrumentalists 61.19, *SD* = 10.57, and for non-musicians/non-singers 52.22, *SD* = 7.69.

**Figure 2:**
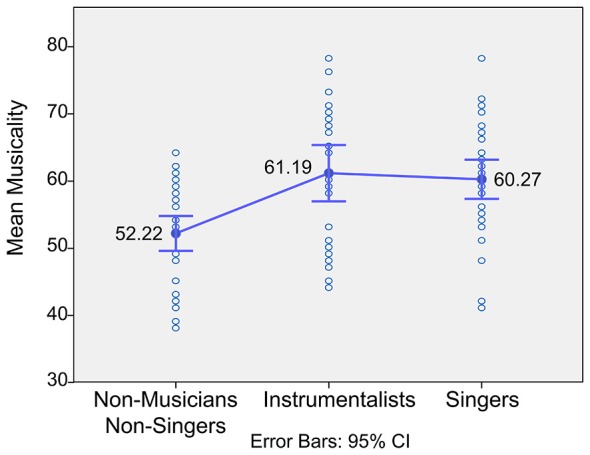
**ANOVA AMMA musicality test.** This figure shows the differences in the performance of the perceptual musicality test (AMMA). Instrumentalists and singers are significantly better than non-musicians/non-singers. However, there is no significant difference between instrumentalists and vocalists on the perceptual musicality test.

1. There was a significant trend, *F*_(2,56.81)_ = 11.52, *p* < 0.01, *ω* = 0.41, indicating that instrument playing and singing capacity heightens the perceptual abilities
2. Planned contrasts revealed that vocalists are significantly better in discriminating musical statements than non-musicians/non-singers *t*_(65.45)_ = 4.20, *p* < 0.01 (one-tailed). Just like vocalists, instrumentalists are also significantly better in discriminating musical statements compared to non-musicians/non-singers *t*_(45.41)_ = 3.73, *p* < 0.01 (one-tailed). However, there was no significant difference of the perceptual abilities between instrumentalists and vocalists (see Figure 2).

## Discussion

Results, indeed, revealed that vocalists outperform instrumentalists on language imitation tasks of unfamiliar utterances. The present investigation supports that instrumentalists and vocalists both are statistically more sensitive to perceive and discriminate rhythmical and tonal changes of melodies than non-musicians/non-singers. Their heightened ability to perceive musical stimuli, however, can only partly explain why numerous studies reported a positive relationship of musical expertise and oral language acquisition processes. Based on this study, it is virtually impossible to distinguish instrumentalists and vocalists on their ability to perceive musical stimuli while, on the other hand, results clearly indicate that an ability to reproduce unintelligible and unfamiliar utterances adequately is significantly higher in vocalists than in instrumentalists. This supports that vocalists and instrumentalists should be dealt with as different categories in the field of language acquisition research. Furthermore, it stresses that the positive relation between musical talent and language talent in the realm of speech imitation is not reliant on audition alone but also on oromotor induced functional processes.

### Musicians’ Enhanced Perception

This study’s results are consistent with previous investigations on phonetic aptitude and musical expertise (e.g., Schön et al., 2004; Thompson et al., 2004; Wong and Perrachione, 2007; Pastuszek-Lipinska, 2008; Milovanov, 2009; Nardo and Reiterer, 2009; Reiterer et al., 2011; Hu et al., 2012; Christiner and Reiterer, 2013; Martínez-Montes et al., 2013). Musicians (instrumentalists and vocalists) were better in imitating unintelligible speech when compared to non-musicians/non-singers. Furthermore, the ability to imitate unintelligible speech is significantly higher in people with higher musical aptitude, even though the language tested (Hindi) was rhythmically differently organised and contains non-native language features (e.g., retroflex sounds) which are largely unknown to German participants. The underlying mechanisms for musicians’ better performance in speech imitation have been discussed in much detail in the recent literature and approached from various angles. Research in neuroscience, for instance, concluded that musicians possess a better working memory (e.g., Koelsch et al., 2009; Rota and Reiterer, 2009; Schulze et al., 2011; Rodríguez-Fornells et al., 2012; Schulze and Koelsch, 2012) and have anatomical endowments in the brain (Schneider et al., 2002; Kleber et al., 2010) which differentiates them from average people to name but a few. Behavioral research, on the other hand, found that musicians may treat short and unintelligible speech streams like musical statements (Christiner and Reiterer, 2013), or rely on the “musical components of speech” when listening to new utterances (Milovanov, 2009). Others demonstrated that musicians could incorporate new utterances more easily (Kraus and Chandrasekaran, 2010) and remember longer sound chunks (Pastuszek-Lipinska, 2008), leaving no doubt about the positive transfer of musical abilities on foreign language perception.

### The Vocal Motor System and its Neural Underpinnings

The present results point to statistically significant differences between instrumentalists and vocalists in oral language imitation abilities, although both are perceptually indistinguishable according to this study’s musicality measurements (AMMA). If the difference does not lie in their perceptual musical abilities, it can only concern the vocal motor ability and its effect on language functions. The outperforming of vocalists over instrumentalists shows that the oromotor system plays a crucial role in language acquisition processes. In this study the Hindi stimuli were selected because they contain retroflex consonants which are rather uncommon and difficult to be produced by native German speakers. Singers’ better performances indicate that singing and speech production are based on the same principles (García-López and Gavilán Bouzas, 2010). Both vocal behaviors are largely bihemispherically, cortically and subcortically organised (Özdemir et al., 2006; Ackermann, 2008; Grabski et al., 2012a; Ziegler and Ackermann, 2013; Ackermann et al., 2014) and draw on common grounds.

Being able to imitate foreign accents on a native level is a highly valued ability. This requires the speaker’s awareness of language specific temporal, dynamic and tonal specialities. When acquiring a language, beginners, however, most often fail to understand where a word begins or ends in a speech stream. Thus, language learners apply the same segmentation strategy of their mother tongue to that of the foreign language (Patel, 2008). Language learners, therefore, need to adapt intonation, word stress, rhythm and melodic aspects of the target language to be as accurate as possible. This requires an enhanced perceptual ability which has been the dominant view in the recent literature, although, controversially, the importance of somatosensory information has been reported as well (e.g., Nasir and Ostry, 2009). However, in how far vocal motor ability and vocal motor flexibility play a major cause has poorly been investigated. Recent research showed that the changes of the vocal motor commands lead to perceptual shifts (Nasir and Ostry, 2009) demonstrating that language production and perception are ultimately linked with each other. Some decades ago Liberman already introduced the motor theory of speech perception and proposed that speech perception of an utterance is “to perceive a specific pattern of intended gesture” (Liberman and Mattingly, 1985). Broadly speaking, this would mean that acoustic speech sounds are transformed gestures and predicate that speech perception and production are “in two ways motor” (Liberman and Mattingly, 1985). Another more recent study provides evidence that the oromotor system may be involved in laying down memory (Schulze et al., 2012). This links speech perception, production and memory and would also explain why vocalists with their refined vocal ability can outperform instrumentalists despite their same perceptual musical ability. The vocal motor system assists memorization and an enhanced vocal flexibility may speed this process up.

Vocal motor training, however, has also shown to affect brain structure. While structural changes in the brain caused by developmental factors through language use has received considerable attention (e.g., Dubois et al., 2006; Brauer et al., 2011), an equivalent research for the influence of singing on brain development does not exist. Brain research in maturational language studies showed particular interest in how the connections between the arcuate fascicle, superior longitudinal fasciculus and the extreme capsulate fiber system influences language acquisition processes (Brauer et al., 2011). Within this study the arcuate fasciculus is most important as its role in motor execution of vocalization is well-known (Basser, 1995). Vocal motor induced alterations of the brain have been found recently in “the dorsal and ventral branches of the left AF” in vocalists which differentiate them from pure instrumentalists and non-musicians (Halwani et al., 2011) and may be one marker for higher vocal ability. In the present study singers were trained and had several years of training. It could be speculated that the years of vocal-motor training lead to structural changes and to higher connectivity between the auditory cortex and the somatosensory cortex. This has been supported recently as song-like training leads to structural adaptations in the arcuate fasciculus of people who suffer from brain lesions after successful speech recovery which improves spontaneous speech production (Schlaug et al., 2009). This speech therapy, the *Melodic Intonation Therapy* (MIT), uses similar tools as singing instruction including syllable lengthening and intoning (singing; Norton et al., 2009). The reasons why the *MIT* improves the spontaneous speech production in aphasics, have not been fully understood yet. A recent study suggests that rhythm may be most relevant in speech recovery (Stahl et al., 2011), while others favour the intoned instructions as a whole (e.g., Schlaug et al., 2009). But while auditory influence on speech recovery has received considerable attention, vocal motor induced anatomical adaptations in the brain have been largely ignored. It might even be more likely that the improvement in the spontaneous speech production of aphasics is a combination of more processes. In how song-like instruction play a role may be difficult to explain. However, singers’ higher ability to imitate speech is reliant on their flexible vocal motor ability which resembles the flexibility of infants who can virtually learn all languages and phonemes within a particular time window (Kuhl, 2004). However, it may also be input dependent. Language targeted at infants is usually more song like in its quality (Murphey, 1990) and more musical (Brandt et al., 2012). Pre-speech research has also demonstrated that early vocalizations of infants “contains melodic constituents for both musical and prosodic structures” (Wermke and Mende, 2009). Singing integrates flexible vocal motor training and music-like instructions which may simulate a language learning situation analogous to infants’ L1 learning and it may therefore be activating one of the most important language acquisition devices.

## Conclusion

Based on this study, it is virtually impossible to distinguish instrumentalists and vocalists on their ability to perceive musical stimuli while, on the other hand, results clearly indicate that an ability to imitate foreign speech (speech production) is significantly higher in vocalists than in instrumentalists. This indicates that vocalists and instrumentalists should be regarded as individual categories in language acquisition research and not summed up under the term “musicians”. According to this study, adaptive plasticity for speech imitation relies equally on production and perception. Vocal flexibility of singers, on the other hand, has a positive transfer effect to the imitation of new and unusual utterances demonstrating that singing and speech imitation/production are closely connected. The present findings, however, also have implications on language learning and teaching. In the light of the present findings it may be justified to rethink language teaching as such. As shown in this research, musical expertise enhances language functions in adults, which shows that language teaching might benefit if musical input is included in language teaching.

## Conflict of Interest Statement

The authors declare that the research was conducted in the absence of any commercial or financial relationships that could be construed as a potential conflict of interest.
